# Supplementary material for: HIVprotI: an integrated web based platform for prediction and design of HIV proteins inhibitors
Source: J Cheminform. 2018 Mar 9;10:12. doi: 10.1186/s13321-018-0266-y (PMC5845081; doi:10.1186/s13321-018-0266-y)
Supplement: Supplementary file 2 — Additional file 2. Source code of HIVProtI web server. [file 13321_2018_266_MOESM2_ESM.zip › HIVprotI_Source-code/hivprotI/weka-3-6-6/doc/weka/classifiers/functions/neural/SigmoidUnit.html]

SigmoidUnit


---


|  |  |  |
| --- | --- | --- |
| **Overview** \| **Package** \| **Class** \| **Tree** \| **Deprecated** \| **Index** \| **Help** \| **Weka's home** | |  |
| **PREV CLASS**   NEXT CLASS | **FRAMES**    **NO FRAMES**     **All Classes** |  |
| SUMMARY: NESTED \| FIELD \| CONSTR \| METHOD | DETAIL: FIELD \| CONSTR \| METHOD |  |


---


## weka.classifiers.functions.neural Class SigmoidUnit

```
java.lang.Object
  weka.classifiers.functions.neural.SigmoidUnit
```

**All Implemented Interfaces:**: java.io.Serializable, NeuralMethod, RevisionHandler

---

``` public class SigmoidUnit extends java.lang.Object implements NeuralMethod, RevisionHandler ```

This can be used by the
neuralnode to perform all it's computations (as a sigmoid unit).

**Version:**
:   $Revision: 1.7 $

**Author:**
:   Malcolm Ware (mfw4@cs.waikato.ac.nz)

**See Also:**: Serialized Form

---

**Constructor Summary**

`SigmoidUnit()`


| **Method Summary** | |
| --- | --- |
| `double` | `errorValue(NeuralNode node)`             This function calculates what the error value should be. |
| `java.lang.String` | `getRevision()`             Returns the revision string. |
| `double` | `outputValue(NeuralNode node)`             This function calculates what the output value should be. |
| `void` | `updateWeights(NeuralNode node, double learn, double momentum)`             This function will calculate what the change in weights should be and also update them. |

**Methods inherited from class java.lang.Object**

`equals, getClass, hashCode, notify, notifyAll, toString, wait, wait, wait`

**Constructor Detail**

### SigmoidUnit

```
public SigmoidUnit()
```


**Method Detail**

### outputValue

```
public double outputValue(NeuralNode node)
```

:   This function calculates what the output value should be.

    :   **Specified by:**: `outputValue` in interface `NeuralMethod`
    :   **Parameters:**: `node` - The node to calculate the value for. **Returns:**: The value.

---


### errorValue

```
public double errorValue(NeuralNode node)
```

:   This function calculates what the error value should be.

    :   **Specified by:**: `errorValue` in interface `NeuralMethod`
    :   **Parameters:**: `node` - The node to calculate the error for. **Returns:**: The error.

---


### updateWeights

```
public void updateWeights(NeuralNode node,
                          double learn,
                          double momentum)
```

:   This function will calculate what the change in weights should be
    and also update them.

    :   **Specified by:**: `updateWeights` in interface `NeuralMethod`
    :   **Parameters:**: `node` - The node to update the weights for.: `learn` - The learning rate to use.: `momentum` - The momentum to use.

---


### getRevision

```
public java.lang.String getRevision()
```

:   Returns the revision string.

    :   **Specified by:**: `getRevision` in interface `RevisionHandler`
    :   **Returns:**: the revision


---


|  |  |  |
| --- | --- | --- |
| **Overview** \| **Package** \| **Class** \| **Tree** \| **Deprecated** \| **Index** \| **Help** \| **Weka's home** | |  |
| **PREV CLASS**   NEXT CLASS | **FRAMES**    **NO FRAMES**     **All Classes** |  |
| SUMMARY: NESTED \| FIELD \| CONSTR \| METHOD | DETAIL: FIELD \| CONSTR \| METHOD |  |


---
